# Neopterin as a Marker of Response to Antiviral Therapy in Hepatitis C Virus Patients

**DOI:** 10.1155/2012/619609

**Published:** 2012-06-28

**Authors:** Gregory F. Oxenkrug, Pura J. Requintina, Dennis L. Mikolich, Robin Ruthazer, Kathleen Viveiros, Hannah Lee, Paul Summergrad

**Affiliations:** ^1^Psychiatry and Inflammation Program, Department of Psychiatry, Tufts Medical Center, Tufts University, Boston, MA 02459, USA; ^2^Institute for Clinical Research and Health Policy Studies, Tufts Medical Center, Tufts University, Boston, MA 02459, USA; ^3^Hepatology Clinic, Division of Gastroenterology, Department of Medicine, Tufts Medical Center, Tufts University, Boston, MA 02459, USA

## Abstract

Predicting the efficacy of antiviral treatment of hepatitis C virus (HCV) is of importance for both patient well-being and health care expense. The expression of interferon-stimulated genes (IFN-SGs) in the liver was suggested as a marker of response to anti-viral therapy. IFN-SGs encode the guanosine triphosphate cyclohydrolase 1 (GTPCH), a rate-limiting enzyme of pteridines biosynthesis. Neopterin, a stable byproduct of GTPCH-catalyzed reaction, is used as a marker of interferon-induced GTPCH activation. We hypothesized that assessment of neopterin concentrations might predict the response to antiviral therapy. Neopterin concentrations were evaluated in 260 HCV patients treated by pegylated interferon combined with ribavirin. Mean and median pretreatment neopterin concentrations were lower in patients with sustained virological response than in nonresponders. The rate of response was twofold higher among patients with pretreatment neopterin levels <16 nmol/L than in patients with neopterin levels ≥16 nmol/L, even after controlling for HCV genotype status. Our study suggests that the pretreatment level of neopterin might be used in routine clinical practice as rapid and cost-effective marker to predict the response to antiviral therapy in HCV patients.

## 1. Introduction

Hepatitis C virus (HCV) is the most common blood-borne infection and a major cause of chronic liver disease, cirrhosis, and primary hepatocellular carcinoma and one of the leading indications for liver transplant [1]. 

The current standard therapy for chronic HCV (pegylated-interferon- (pegIFN-) *α* combined with ribavirin) has limited efficacy (about 50%), is costly, and involves severe medical and psychiatric side effects. Recently introduced protease inhibitors, Telaprevir and Boceprevir, are effective in HCV1 and HCV2 while their antiviral activity is limited in HCV3 and HCV4 [2]. Therefore, search for biological markers to predict the response to antiviral treatment is of importance for both patient well-being and health care expense. HCV genotypes predict more favorite response among HCV1 and HCV4 (in comparison with HCV2- and HCV3- infected patients [1, 2]). The value of currently used assessment of allelic variants of the IL28B gene encoding IFN*λ*3 predicts antiviral response in HCV1 and HCV4 and is attenuated, but relevant also in HCV2 and HCV3, especially in patients not achieving rapid virological response [3]. Assessment of expression of interferon-stimulated genes (IFN-SGs) in the liver (but not in plasma) predicted antiviral response independently from IL28B polymorphism [4, 5]. These studies suggest the inverse correlation between antiviral response and concentrations of proteins production of which is encoded by IFN-SGs.

IFN-SGs induce rate-limiting enzyme of pteridines biosynthesis, guanosine triphosphate cyclohydrolase I (GTPCH). IFN-induced activation of GTPCH in humans might be assessed by measuring of plasma, serum, saliva, urine, and spinal fluids concentrations of neopterin, a stable water-soluble byproduct of the first and rate-limiting reaction of pteridines biosynthesis catalyzed by GTPCH [6].

The present study explored the possibility that pretreatment plasma neopterin concentrations (as a marker of the expression of the IFN gene that encodes GTPCH) predict the response to antiviral therapy by pegIFN*α* combined with ribavirin.

## 2. Methods

### 2.1. Subjects

Neopterin concentrations were evaluated in 260 HCV patients treated by peginterferon- (IFN-) alpha (Pegasys or PegIntron) (subcutaneous injections, 120 to 180 *μ*g/week) in combination with ribavirin (1,000 to 1,200 mg/day). Doses were determined by the patients' body weight. Treatment lasted for 6–12 months depending on the virus genotype. The study was approved by the Tufts Medical Center IRB, and written consents were obtained for participation in the study. 

### 2.2. Measurement of Neopterin

Blood was drawn into EDTA tubes. Blood samples were protected from light, centrifuged to obtain plasma, and frozen at −80°C before blinded assay by sandwich ELISA using commercially available kits (American Research Products, Inc., Belmont, MA) according to the manufacturer instructions. Duplicate measurements were performed for each undiluted sample. Neopterin concentrations were reported as nanomoles per liter (nmol/L) [7].

### 2.3. Definition of Response to Therapy

The sustained virological response (SVR) was defined as undetectable serum HCV RNA (<51 IU/mL) 24 weeks after completion of treatment. Patients were considered nonresponders if they had only a minimal decrease of <2 log_10_ IU from their baseline levels after completion of 24 weeks of treatment. 

### 2.4. Statistical Analysis

Patient characteristics, including gender, race, and HCV status, were compared between SVR and nonresponders using chi-square tests. Since distribution of neopterin levels was found to be highly skewed, neopterin concentrations were summarized with the median and 25th to 75th percentiles, and the distribution of neopterin was compared between SVR and nonresponders using the Wilcoxon rank-sum test. Logistic regression was used to compute unadjusted odds ratios and 95% confidence intervals for the relationship of neopterin and response and for HCV status and response. Multiple logistic regression was also used to test for an interaction between neopterin level and HCV status and estimate adjusted odds ratios of neopterin and response for each HCV subgroup. Considering that neopterin plasma concentration higher than 16 nmol/L predicted high mortality rate in 6-year follow-up study of community dwellers [7], neopterin was categorized as less than versus at least 16 nmol/L for the logistic regression analyses to simplify the interpretation of the results. Analyses were done using the SAS System for Windows version 9.2 (Copyright © 2002–2008 by SAS Institute Inc., Cary, NC, USA).

## 3. Results

Average age of all participants was 53.3 ± 8.7 years. There were more men (70%) than women among 260 study patients. 83% of patients were American Caucasians and 27% Afro-Americans, Hispanic, and Asians. As expected from the literature, the response rate was higher in patients with HCV2 and HCV3 genotypes (57%) than in patients with HCV1 and HCV4 genotypes (27%) (unadjusted odds ratio 3.6, 95% CI: 1.8–6.7; *P* <0.0002).

There were no gender or race differences between SVR and nonresponders. Responders were slightly younger (51.8 ± 9.6) than nonresponders (54.1 ± 8.2) (*P* = 0.04).

There were no differences in plasma neopterin concentrations between females (median (Q1–Q3) = 20.8 (13.8–36.8)) and males (median (Q1–Q3) = 19.2 (11.9–34.8)) (*P*-value = 0.21, Wilcoxon rank-sum test).

Mean and median neopterin concentrations were higher in nonresponders than in SVR (Table 1).

The overall rate of response to antiviral treatment in studied patients was lower (32%) than reported in the literature (50%). Response rate correlated with neopterin levels. Among patients with neopterin levels of <16 nmol/L response rate was 51% for all genotypes, 42% for HCV1 and HCV4 subset, and 74% for HCV2 and HCV3 subset. Patients with neopterin levels >16 nmol/L had the lowest rate of response (21% for all genotypes, 19% for HCV1 and HCV4 subset, and 38% for HCV2 and HCV3 subset) (Table 2).

Therefore, both neopterin concentration and HCV genotype emerged as factors that appear to have (unadjusted) associations (*P*< 0.5) related to response to antiviral therapy.

Our data was further analyzed by a multivariable logistic regression model with neopterin (categorized at <16 versus ≥16 nmol/L), HCV genotype (subset of HCV1 and HCV4 versus HCV2 and HCV3), and the interaction term.

Antiviral response rate in patients with neopterin concentrations of <16 nmol/L was about twofold higher than in patients with neopterin concentrations of ≥16 nmol/L for all genotypes and for the subsets of HCV1 and HCV4 patients and HCV2 and HCV3 patients (Table 2). There was no difference between odds ratios for HCV1 and HCV4 versus HCV2 and HCV3 patients (*P* = 0.61).

## 4. Discussion

To the best of our knowledge this is the first observation of a negative relationship between the pretreatment neopterin concentrations and response to antiviral therapy. Mean and median pretreatment neopterin concentrations were higher in nonresponders than in SVR patients. Multivariable modeling revealed that rate of response to treatment was twofold higher among patients with pretreatment neopterin levels <16 nmol/L than in patients with pretreatment neopterin levels ≥16 nmol/L, even after controlling for HCV genotype status.

The negative relationship between pretreatment neopterin and response to antiviral therapy was not observed previously because (most likely) of rather small number of studied patients [8].

Neopterin concentrations in humans reflect the activity of GTPCH, the enzyme encoded by IFN-SGs [6]. High pretreatment neopterin plasma concentrations might suggest the preactivation of the endogenous IFN system induced by chronic HCV infection. One of the causes of a resistance to antiviral therapy in patients with high pretreatment neopterin concentrations might be the refractory state of the preactivated endogenous IFN signaling pathways to further stimulation by antiviral therapy [4, 5].

Since our study was not designed to include patients naive to IFN-alpha treatment, they were exposed to both HCV and IFN-alpha. Combined exposure to HCV and IFN-alpha might upregulate their IFN system. Thus, there were almost two times more patients in HCVI and HCV4 subset with neopterin levels >16 nmol/L than with <16 nmol/L. The rate of response in patients with neopterin levels lower than 16 nmol/L was in line with the literature data (50%). The prediction of the response to antiviral treatment could become important for future therapies with HCV protease or polymerase inhibitors because patients with a preactivated endogenous IFN system would be exposed to direct antivirals without an effective protection against resistance development provided by coadministration of pegIFN-*α*/ribavirin [2, 5]. Considering the wide use of IL28b polymorphism for predicting of antiviral response, future studies of correlation between IL28B and neopterin test might be of importance for clinical practice.

## 5. Conclusions

The results of our study suggest that the evaluation of pretreatment concentrations of neopterin might be used as a marker to predict the response to antiviral therapy in HCV patients. A “neopterin test” is cost-effective, helpful in both HCV1 and HCV4 and HCV2 and HCV3 genotypes, and less invasive than liver biopsy (in comparison with genotype analyses of liver IFN-SGs) [4, 5].

## Figures and Tables

**Table 1:** Neopterin concentrations in HCV responders and nonresponders to antiviral therapy.

	All patients	Responders	Nonresponders	*P* value
Neopterin (nmol/L)	*N* = 260	*N* = 84	*N* = 176	
Mean ± standard deviation	28.8 ± 26.4	22.8 ± 24.8	31.4 ± 27.2	0.015 (Student's *t*-test)
Median [25th–75th percentile]	19.4 [12.2–36.4]	13.0 [9.4–25.7]	24.4 [15.0–26.8]	<.0001 (Wilcoxon rank-sum test)

**Table 2:** Neopterin and response rates to antiviral therapy in HCV patients (*N* = 260).

Neopterin (nmol/L) (*N* ^∗^)	Response rate	Odds ratio (95% CI)	Chi-square and *P* value
All genotypes:			
<16 (*N* = 97)	51%	3.73 (CI: 2.16–6.45)	Chi = 23.14 (df = 1), *P* = <.0001
≥16 (*N* = 163)	21%		
HCV 1 or 4:			
<16 (*N* = 73)	42%	3.20 (CI: 1.6–6.0)	Chi = 32.5 (df = 3), *P* = <.0001
≥16 (*N* = 133)	19%		
HCV 2 or 3:			
<16 (*N* = 29)	74%	4.60 (CI: 1.2–16.60)	Chi = 32.5 (df = 3), *P* = <.0001
≥16 (*N* = 25)	38%		

^∗^N: number of observations.
